# Effects of vitamin D supplementation in patients with rheumatoid arthritis: A systematic review and meta-analysis

**DOI:** 10.1016/j.heliyon.2025.e42463

**Published:** 2025-02-04

**Authors:** Mahsa Ranjbar, Mehran Rahimlou, Maryam Fallah, Kurosh Djafarian, Hamed Mohammadi

**Affiliations:** a Department of Clinical Nutrition, School of Nutritional Sciences and Dietetics, Tehran University of Medical Science, Tehran, Iran; b Students' Scientific Research Center, Tehran University of Medical Sciences, Tehran, Iran; c Department of Nutrition, Faculty of Medicine, Zanjan University of Medical Sciences, Zanjan, Iran; d Neuroscience Institute, Sports Medicine Research Center, Tehran University of Medical Sciences, Tehran, Iran

**Keywords:** Vitamin D supplementation, Rheumatoid arthritis, Disease activity score 28, C-reactive protein, Erythrocyte sedimentation rate, Serum vitamin D, Visual analog scale

## Abstract

**Background:**

Rheumatoid arthritis (RA) is known as an inflammatory illness. Evidence shows that Vitamin D modulates immunologic function and inflammation by affecting various immunological cells. We decided to run a systematic review and meta-analysis to investigate the relationship between vitamin D supplementation and the outcomes of adult patients suffering from RA.

**Methods:**

We searched electronic databases, using specific search terms in PubMed, Scopus, and ISI Web of Science, until May 2024. Clinical studies involving patients with RA were included if they compared the effects of vitamin D supplementation to either a placebo or standard care. The results from the selected studies were presented as weighted mean differences (WMD) along with a 95 % confidence interval (CI).

**Results:**

Inclusion criteria have been met by 11 studies and presented as part of this analysis. The results indicate a major influence of vitamin D supplementation on the Disease Activity Score 28 (DAS-28) (WMD: −0.83, 95 % CI: −1.38 to −0.28, p-value <0.001), C-reactive protein (CRP) level (WMD: −0.24, 95 % CI: −0.45 to −0.03, p-value = 0.03), erythrocyte sedimentation rate (ESR) level (WMD: −4.08, 95 % CI: −4.67 to −3.50, p-value <0.001), serum vitamin D level (WMD: 12.69, 95 % CI: 1.80 to 23.59, p-value = 0.02), and non-significant effect on the health assessment questionnaire (HAQ) and visual analog scale on pain (VAS-pain) scores. Grades of Recommendation, Assessment, Development, and Evaluation (GRADE) assessment shows moderate certainty of the evidence for all outcomes except for serum vitamin D, which has a high certainty of the evidence.

**Conclusion:**

To improve DAS-28, CRP, ESR, and serum vitamin D in RA patients, vitamin D supplements may be beneficial, although the optimal dosage and length of treatment are still unknown.

## Highlights

• Significant improvements were observed in DAS-28 scores, CRP, ESR, and Serum vitamin D levels.
• No significant effects were found on the HAQ and VAS-pain scores.
• GRADE assessment indicated moderate certainty for most outcomes, with high certainty for serum vitamin D levels.
• Vitamin D supplementation may benefit RA patients, but optimal dosage and treatment duration remain unclear.

## 1 Introduction

Rheumatoid arthritis (RA) is an autoimmune disorder that falls in the category of chronic rheumatic illnesses [1]. It has been estimated that the prevalence of RA is nearly 1 % around the world [2]. While the precise etiology of RA remains unknown, there are pieces of evidence that it is a mixture of genetic predispositions and environmental factors [3]. The most commonly affected areas in RA are the joints, leading to symptoms such as pain, stiffness, and swelling [4]. Nevertheless, RA can also affect other parts of the body, potentially leading to severe complications, including cardiovascular disease and lung issues [5]. Further than these physical impacts, RA can significantly deteriorate both mental health and overall quality of life of patients [6]. The underlying mechanism of RA, like that of other autoimmune diseases, involves an unusual attack by immune system cells on the body's tissues [7]. This immune response triggers the activation of pro-inflammatory cytokines, which contribute to widespread inflammation throughout the body [8]. Management of RA typically involves a combination of therapeutic agents aimed at mitigating inflammation, reducing symptoms, and preventing joint damage [9]. Additionally, emerging evidence suggests that supplementary treatments may also improve symptoms and enhance the overall well-being of RA patients [[10], [11], [12]].

Vitamin D is vital in many aspects, such as bone health and immune function [13]. This vitamin can be obtained in several ways, including exposure to sunlight, foods, and especially supplementation [14]. Vitamin D plays a significant role in regulating the immune system, and its mechanisms of action are particularly relevant in the context of autoimmune diseases [15]. Research has demonstrated that it can modulate inflammatory cytokines [16]. Vitamin D Receptor (VDR) is present in various immune cells, including T cells, B cells, dendritic cells, and macrophages [17]. When calcitriol, the active form of vitamin D, binds to VDR, it initiates genomic and non-genomic signaling pathways that influence immune function [18]. Furthermore, this vitamin promotes regulatory T cells and helps to prevent autoimmune reactions [19]. The literature claims vitamin D is crucial in developing and treating RA [20]. It has been suggested that Vitamin D promotes the differentiation of naive T cells into regulatory T cells (Tregs), which help maintain immune tolerance and inhibit autoreactive T cells that can contribute to RA [20]. Also, it may hinder the production of pro-inflammatory cytokines while promoting anti-inflammatory cytokines, thus helping to reduce systemic inflammation [21]. As a result, because of its immunomodulatory effect, supplementation with vitamin D has been recommended for RA patients to help manage their condition [22].

Some reviews have been examining the impact of vitamin D supplements on the outcomes of these patients, but they have some limitations, as they did not include all eligible articles in 2020 [23], or specify the source of heterogeneity in 2023 [24]. Moreover, there is no detailed subgroup analysis in this field. Because of this, the current study aimed to review the effects of supplementation with vitamin D in adult patients with RA, specify the sources of heterogeneity, and perform detailed subgroup analysis on all eligible studies. The outcomes examined in this study were C-reactive protein (CRP), and erythrocyte sedimentation rate (ESR), both representing the disease activity in RA [25]. Elevated levels of these markers often correlate with active disease, indicating increased inflammation and joint involvement [26]. Disease activity score 28 (DAS-28) also reveals the activity of the disease [27]. Moreover, quality of life was examined by the health assessment questionnaire (HAQ) [28], and pain via visual analog scale (VAS) [29].

## 2 Methods

### 2.1 Strategy of search

**Fig. 1:**
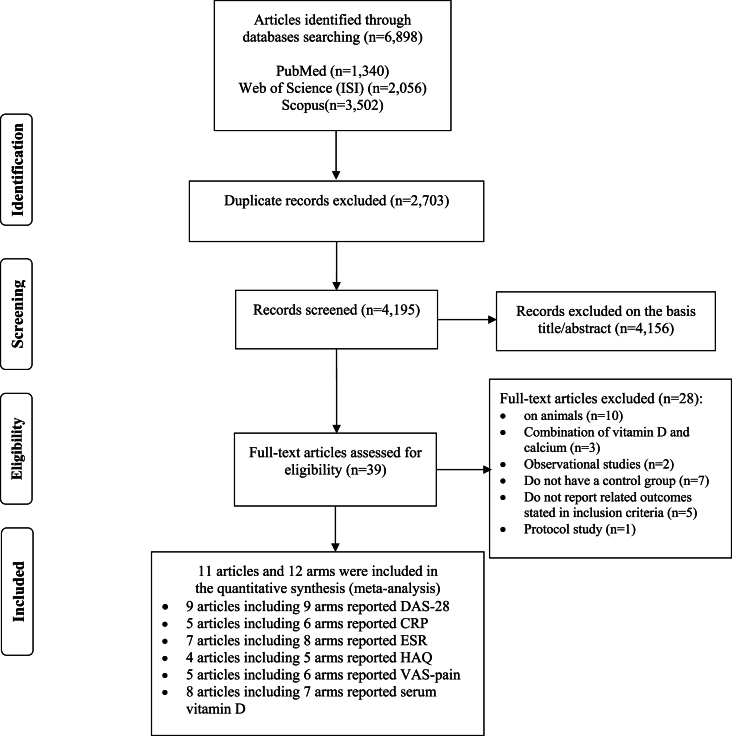
Literature search and review flow diagram for selection of the studies.

The Preferred Reporting Item for Systematic Review and Meta-analysis (PRISMA) guideline was deployed for this study (Fig. 1). A predefined search item was used for the systematic search in PubMed, Scopus, and ISI Web of Science, up to May 2024. The comprehensive search strategy is recorded in Table S1. Title and abstract screening were performed to find eligible studies on the inclusion and exclusion criteria. Two authors (M.R and M.F) cheeked full studies texts for eligibility. If there were any controversies, the third reviewer (H.M) checked it out. Relevant articles were reference-checked to find any missed study.

### 2.2 Eligibility criteria

PICOS (population, intervention/exposure, comparator, outcome, and study design) framework was used for inclusion and exclusion Criteria (Table 1). In this review, inclusion criteria include: 1) 18 and over aged participants; 2) examined the impacts of vitamin D supplementation on patients suffering from RA; 3) reported related outcomes including DAS-28, CRP, ESR, HAQ, score, VAS-pain score, and serum vitamin D; and 4) compared the effects of vitamin D supplementation in intervention and control groups. Studies were excluded if they focused on individuals under 18 years old, as well as pregnant or breastfeeding women.

**Table 1:** PICOS criteria for inclusion and exclusion of studies.

Parameter	Criteria
Population	Adults aged older than 18 suffering from rheumatoid arthritis
Intervention	Vitamin D
Control/comparator	Placebo or nothing
Outcomes	DAS28, CRP, ESR, HAQ, VAS Serum vitamin D
Study design	Controlled trails

Abbreviations: DAS-28, disease activity score; CRP, C-reactive protein; ESR, erythrocyte sedimentation rate; HAQ, the health assessment questionnaire; VAS, visual analog scale.

### 2.3 Data extraction

For the studies deemed eligible, the two independent reviewers (M.R and M.F) extracted information including the author's name and year, study location, design and duration, participant characteristics, intervention details (type and dosage of vitamin D supplementation), information about the control group regarding receive placebo or not, and the necessary results for the included outcomes including DAS-28, CRP, ESR, HAQ, VAS-pain, and serum vitamin D.

### 2.4 Risk of bias and certainty of the evidence

To assess the risk of bias in this study, we used the Cochrane Collaboration tool [30], which was done by two authors (M.R and M.F) in duplicate. The Grades of Recommendation, Assessment, Development, and Evaluation (GRADE) approach was used to determine the certainty of evidence. H.M. resolved discrepancies between the reviewers.

### 2.5 Data synthesis and statistical analysis

Data needed for statistical analysis was gathered from a preformatted Excel table and subsequently input into Stata 14 software (Stata Corp, College Station, TX, USA). The relevant effect size (mean or difference of means) and their standard deviations (SD) were utilized for analysis. When studies reported varying effect sizes, we converted them into a common effect size whenever feasible. We assessed heterogeneity among the studies using Cochran's chi-square test (Q), with the I^2^ statistic indicating the extent of heterogeneity. We employed statistical modeling, sensitivity analyses, and subgroup analyses to identify the sources of heterogeneity in the studies included, depending on the available data from the extracted studies. Publication bias was evaluated using funnel plots, with the relative risk displayed about the inverse of the squared standard error. The assessment of funnel plot asymmetry was conducted using Egger's regression analysis. A sensitivity analysis was done to define the impact of each study or a specific group of studies on the total effect.

The protocol of this study has been registered with PROSPERO under the number: CRD42023427171.

## 3 Results

### 3.1 Selection and identification of studies

Fig. 1 presents the literature search and flow diagram for the study selection. The search in electronic databases yielded 6898 records, of which 2703 were duplicate citations. 4195 citations were screened, and 39 studies were full-text searches. Table S2 shows the reasons for excluding irrelevant articles. Overall, eleven records met the eligibility requirements and were included in the meta-analysis [[31], [32], [33], [34], [35], [36], [37], [38], [39], [40], [41]].

### 3.2 Characteristics of included studies

Table 2 presents the detailed characteristics of the studies included in the analysis. These studies were carried out from 1973 to 2024 and involved a total of 1129 participants, with 634 in the vitamin D group and 475 in the control group. All participants were individuals diagnosed with RA. The location of these studies was China [33,42], Egypt [31,38], France [32], Italy [34], USA [35], Iran [36], India [39,40], Sweden [37], and the UK [41]. The duration of the intervention was six weeks in 1 study [33], eight weeks in 2 studies [38,40], three months in 5 studies [31,34,36,39,41], six months in 1 study [32], and twelve months in 3 studies [35,37,42]. The type and dose of vitamin D used as intervention were calcitriol with a dose of 0.25–0.5 μg per day or vitamin D3 tablets 200–400 IU (international unit) per day [42], 50,000 IU of Vitamin D2 weekly [31], vitamin D ampoules (cholecalciferol 100,000IU) every 2 weeks [32], 50,000 IU per week of 22-oxa-calcitriol in one group and 50,000 IU per week of calcitriol in another group [33], cholecalciferol 50,000 IU weekly [38,41], cholecalciferol 300,000 IU weekly [34], ergocalciferol 50,000 IU three times weekly for four weeks, then 50,000 IU twice monthly for 11 months [35], ergocalciferol 50,000 IU [36] and 60,000 IU weekly [40], 60,000 IU weekly for 6 weeks, followed by 60,000 IU monthly for 3 months [39], and calciferol 100,000 IU per day [37].

**Table 2:** characteristics of included studies.

	First author (Country; year)	Sex	Mean Age (SD) (year)	Type of Disease	Dose	Sample size (Vitamin D/Placebo)	Duration (W)	Intervention		Muscle damage indices
								Type of vitamin D supplement	Control group	
1	Brohult et al. (Sweden; 1973) [37]	Both	52 (12.75)	RA	100,000IU/daily	49 (24/25)	52	Calciferol	Nothing	ESR
2	Buondonno et al. (Italy; 2017) [34]	Both	55 [13]	Early RA	300,000IU/single administration at the baseline	36 (18/18)	12	Cholecalciferol	Nothing	DAS-28, ESR, CRP, HAQ, VAS-pain, serum vitamin D
3	El-Banna et al. (Egypt; 2020) [31]	Both	45.4 (5.53)	RA	50000 IU/weekly	40 (20/20)	12	Ergocalciferol	Nothing	DAS-28
4	Hansen et al. (USA; 2014) [35]	Both	NR	RA	50000 IU/weekly	22 (11/11)	52	Ergocalciferol	Placebo	DAS-28, HAQ, VAS-pain, serum vitamin D
5	Li et al. (China; 2018) [1,33]	Both	50.35 (10.4)	RA	50000 IU/weekly	246 (123/123)	6	Calcitriol	Placebo (Lactose powder)	ESR, CRP, HAQ, VAS-pain, serum vitamin D
6	Li et al. (China; 2018) [2,33]	Both	50.47 (10.67)	RA	50000 IU/weekly	246 (123/123)	6	22-oxa-calcitriol	Placebo (Lactose powder)	ESR, CRP, HAQ, VAS-pain, serum vitamin D
7	Salesi et al. (Iran; 2012) [36]	Both	50 (12.8)	RA	50000 IU/weekly	98 (50/48)	12	Cholecalciferol	Placebo	DAS-28, ESR, VAS-pain, serum vitamin D
8	Soubrier et al. (France; 2018) [32]	Both	59.8 (10.9)	RA	100,000 IU (IV)/every 2 weeks	59 (29/30)	24	Cholecalciferol	Placebo	DAS-28, ESR, CRP
9	Alotalibi et al., (Egypt; 2021) [38]	NR	NR	RA	50,000 IU/weekly	80 (40/40)	8	Cholecalciferol	Nothing	DAS-28
10	Chandrashekara et al. (India; 2015) [39]	Both	48.6 (12.1)	RA	60, 000 IU weekly for 6 weeks, followed by 60,000 IU 1 month	138 (73/65)	12	Cholecalciferol	Nothing	DAS-28, CRP, ESR, VAS, serum vitamin D
11	Mukherjee et al. (India; 2019) [40]	Both	37.7 (12.2)	RA	60,000 IU weekly	150 (75/75)	8	Calcitriol	Nothing	DAS-28
12	Elfituri (The UK; 2024) [41]	Both	47.2 (12.75)	RA	50000 IU/weekly	68 (48/20)	12	Cholecalciferol	Nothing	DAS-28, CRP, ESR, serum vitamin D

**Abbreviations:** SD, standard deviation; NR, not reported; RCT, randomized control trial; RA, rheumatoid arthritis; IU, international unit; IV, intravenous; W, weeks; DAS-28, disease activity score; CRP, C-reactive protein; ESR, erythrocyte sedimentation rate; HAQ, the health assessment questionnaire; VAS, visual analog scale.

### 3.3 Assessment of risk of bias

The risk of bias regarding the blinding of participants and personnel was high in four studies [31,[38], [39], [40]], unclear in one study [41], and low in the remaining studies. For the blinding of outcome assessment, four studies [31,[38], [39], [40]], also exhibited a high risk of bias, while the risk was unclear in other studies. Regarding incomplete outcome data and selective outcome reporting, the studies demonstrated a low or unclear risk of bias. Details of the assessment can be found in Table S3.

### 3.4 Publication bias

Regarding the Egger's regression analysis and funnel plot, no study showed any signs of publication bias for DAS-28 score (p = 0.948, Egger's test), CRP (p = 0.548, Egger's test), ESR (p = 0.372, Egger's test), HAQ score (p = 0.439, Egger's test), VAS-pain score (p = 0.359, Egger's test), and serum vitamin D (p = 0.287, Egger's test).

### 3.5 Effects of vitamin D supplement on DAS-28 score

**Fig. 2:**
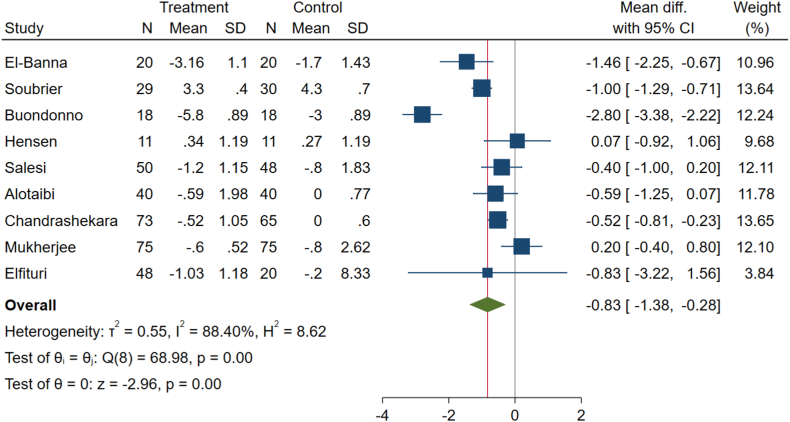
Effects of vitamin D supplement on disease activity score-28 (DAS-28) score of RA patients.

This outcome was assessed in nine arms of clinical trials. The pooled mean differences calculated using the inverse variance method indicated a significant change in the DAS-28 score for these patients (weighted mean difference (WMD): −0.83, 95 % confidence interval (CI): −1.38 to −0.28, p-value <0.001), using a random effect analysis because of considerable between-study heterogeneity (I^2^ = 88.4 %, P < 0.001) (Fig. 2).

### 3.6 Effects of vitamin D supplement on CRP level

**Fig. 3:**
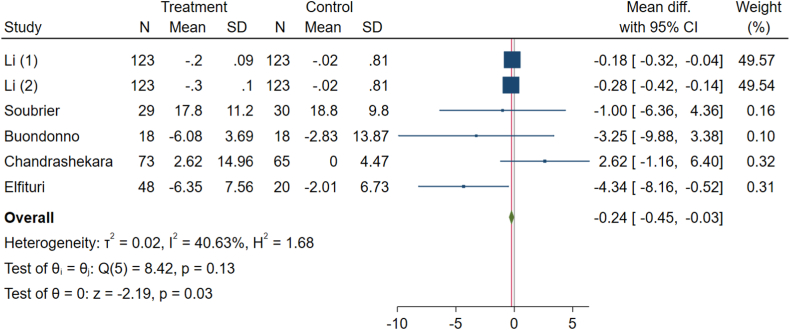
Effects of vitamin D supplement on C-reactive protein (CRP) level of RA patients.

Five clinical trials, encompassing six effect sizes, have evaluated the impact of vitamin D on CRP levels in patients with RA. The pooled mean differences calculated using the inverse variance method reveal a significant change in these patients' CRP levels (WMD: −0.24, 95 % CI: −0.45 to −0.03, p-value = 0.03) (Fig. 3). No significant heterogeneity between the studies was observed (I^2^ = 40.63 %, P = 0.013).

### 3.7 Effects of vitamin D supplement on ESR level

**Fig. 4:**
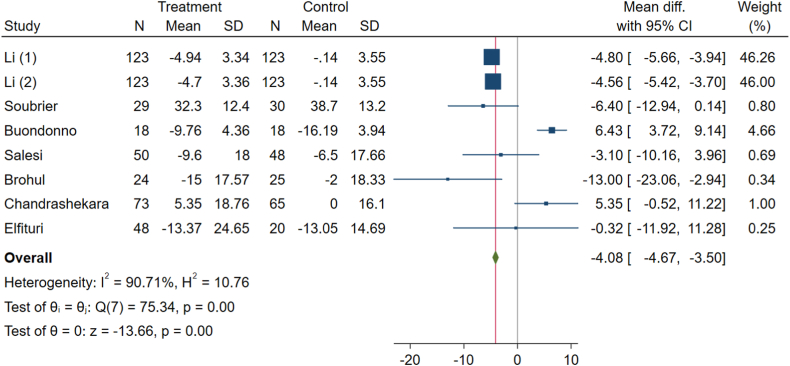
Effects of vitamin D supplement on erythrocyte sedimentation rate (ESR) level of RA patients.

Vitamin D significantly influenced the ESR levels in patients with RA (WMD: −4.08, 95 % CI: −4.67 to −3.50, p-value <0.001) across eight effect-size arms (Fig. 4). A random effect analysis was also used because of considerable heterogeneity among the studies (I^2^ = 90.71 %, P < 0.001).

### 3.8 Effects of vitamin D supplement on HAQ score

**Fig. 5:**
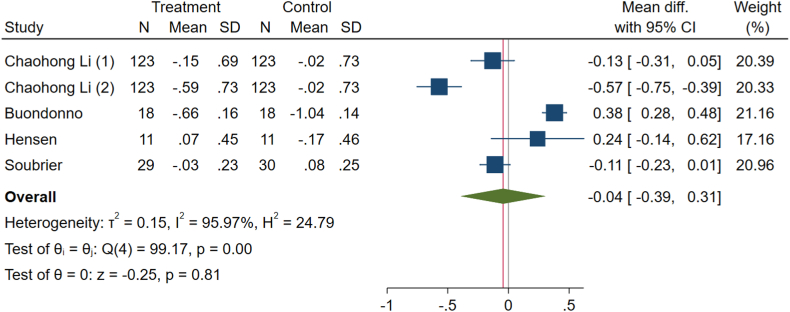
Effects of vitamin D supplement on health assessment questionnaire (HAQ) score of RA patients.

Due to significant variability among the studies (I^2^ = 95.97 %, P < 0.001), a random effects analysis was conducted. Our analysis of five effect size arms indicated that vitamin D did not significantly impact the HAQ score of patients with RA (WMD: −0.04, 95 % CI: −0.39 to 0.31, p-value = 0.81), as illustrated in Fig. 5.

### 3.9 Effects of vitamin D supplement on VAS pain score

**Fig. 6:**
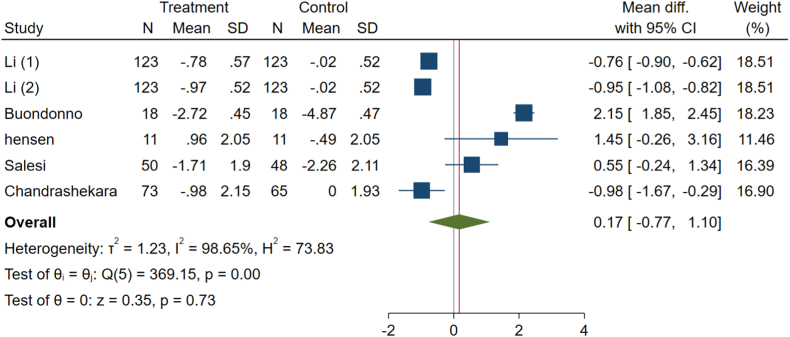
Effects of vitamin D supplement on visual analog scale (VAS) pain score of RA patients.

Our analysis suggests that vitamin D did not have a meaningful effect on the VAS pain scores of patients with RA (WMD: 0.17, 95 % CI: −0.77 to 1.1, p-value = 0.73), based on data from six effect size arms (Fig. 6). Additionally, significant heterogeneity among the studies (I^2^ = 98.65 %, P < 0.001) prompted a random effects meta-analysis.

### 3.10 Effects of vitamin D supplement on serum vitamin D level

**Fig. 7:**
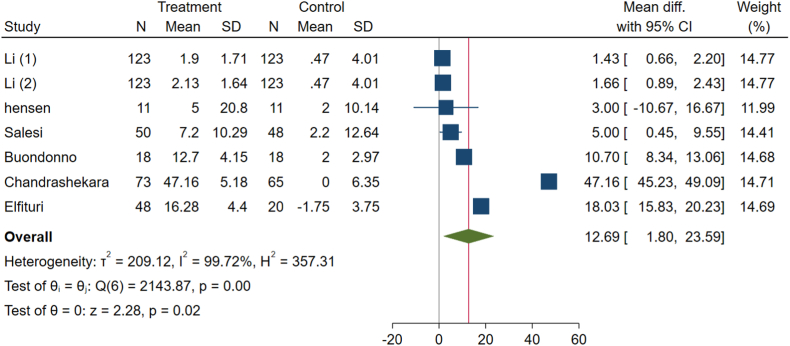
Effects of vitamin D supplement on serum vitamin D level of RA patients.

Our meta-analysis, which examined five groups of effect sizes, indicates that vitamin D significantly influences serum vitamin D levels in patients with RA (WMD: 12.69, 95 % CI: 1.80 to 23.59, p-value = 0.02) (Fig. 7). Additionally, there was a high level of heterogeneity among the studies (I^2^ = 99.72 %, P < 0.001), prompting the use of a random effects meta-analysis.

### 3.11 Sensitivity analysis

Fig. S3 presents the forest plots of sensitivity analysis with a leave-one-out meta-analysis. The sensitivity analysis for DAS-28, ESR, and HAQ demonstrated that removing any study did not impact the overall outcomes, but for CRP, the study by Li et al., for the VAS score study by Buondonno et al., and for the serum vitamin D study by Li et al., both Calcitriol and 22-oxa-calcitriol interventions influence the results of the total outcomes.

### 3.12 Subgroup analysis

The results of the subgroup analysis are presented in Table 3. For DAS-28, subgroups including the low and high risk of bias, developed countries, 12 weeks duration, intake cholecalciferol, all doses, and nothing intake by the control group had a significant impact similar to the total results (p-value <0.05 for all). The heterogeneity was high in all subgroups except for the intervention dose lower than 50,000 IU weekly subgroups (p-heterogeneity: 0.152). Regarding CRP, low risk of bias, both developed and developing countries, <12 weeks duration, calcitriol intervention, and dose of intervention lower than 50,000 IU weekly were the subgroups with significant effects on decreasing CRP level (p-value <0.05 for all) similar to total results. The heterogeneity of CRP was not significant (p-heterogeneity: 0.105). Subgroup analysis on ESR revealed that the result was not significant, with just some concerns in risk of bias, developed country, and 12-week intervention duration subgroups (p-value >0.05 for all). The potential source of heterogeneity for this outcome seems to be the intervention dose of more than 50,000 IU weekly subgroups (p-heterogeneity: <0.001). HAQ scores have significant changes in developed countries, 22-oxa-calcitriol intake, the dose of intervention of more than 50,000 IU weekly, and nothing as placebo subgroups (p-value: <0.05 for all) opposite to the total results. The total result for VAS was insignificant, similar to all risks of bias, 12 weeks and more duration of intervention, cholecalciferol ergocalciferol intake, the dose of intervention of more than 50,000 IU weekly, and nothing given as placebo subgroups (p-value: >0.05 for all). Due to these results, the source of heterogeneity for HAQ and VAS may be related to the developing countries subgroup (p-heterogeneity: <0.001). Most serum vitamin D levels subgroups have improved significantly, similar to the total results. Some concerns and low risk of bias, developing countries, more than 12 weeks, intake of ergocalciferol, and dose of intervention of more than 50,000 IU weekly subgroups show insignificant results (p-value: >0.05 for all). The source of heterogeneity for this outcome may be due to the 12 weeks of intervention and nothing given as placebo subgroups (p-heterogeneity: <0.001 for both).

**Table 3:** Pooled estimates of the effects of vitamin D on rheumatoid arthritis in different subgroups.

Group	Comparisons, n	WMD (95 % CI)	P-value	I^2^ (%)	P-heterogeneity
Subgroup analysis for the effect of vitamin D supplementation on DAS-28.					
**Total**	**9**	**−0.83 (-1.38 to -0.28)**	**<0.001**	**88.4**	**<0.001**
**Risk of bias**					**<0.001**
Low	1	−2.8 (−3.38, −2.22)	<0.001	.	.
Some concerns	2	−0.58 (−1.60, 0.45)	0.272	75.6	0.043
High	6	−0.51 (−0.90, −0.12)	0.010	54.7	0.051
**Country**					**0.297**
Developed	4	−1.24 (−2.56, 0.07)	0.064	91.9	<0.001
Developing	5	−0.51 (−0.92, −0.10)	0.015	63.6	0.027
**Duration**					**0.296**
<12 weeks	3	−0.34 (−0.78, 0.11)	0.140	58.2	0.092
12 weeks	4	−1.48 (−2.87, −0.10)	0.036	90.5	<0.001
>12 weeks	2	−0.58 (−1.60, 0.45)	0.272	75.6	0.043
**Type of vitamin D**					**0.023**
Ergocalciferol	2	−0.73 (−2.22, 0.77)	0.342	82.0	0.018
Cholecalciferol	6	−1.05 (−1.72, −0.38)	0.002	90.4	<0.001
Calcitriol	1	0.20 (−0.40, 0.80)	0.517	.	.
**Dose of intervention**					**0.445**
≤50,000 IU/weekly	5	−0.62 (−1.13, −0.12)	0.016	40.4	0.152
>50,000 IU/weekly	4	−1.02 (−1.91, −0.13)	0.024	95.1	<0.001
**Type of control**					**0.429**
Placebo	6	−0.57 (−1.18, 0.04)	0.065	68.7	0.041
Nothing	3	−1.02 (−1.94, −0.09)	0.031	92.0	<0.001
Subgroup analysis for the effect of vitamin D supplementation on CRP.					
**Total**	**6**	**−0.24 (-0.45, -0.03)**	**0.040**	**45.0**	**0.105**
**Risk of bias**					**0.946**
Low	3	−0.23 (−0.33, −0.13)	<0.001	0.0	0.423
Some concerns	1	−1.0 (−6.38, 4.38)	0.715	.	.
High	2	−0.85 (−7.76, 5.97)	0.806	85.9	0.008
**Country**					**0.029**
Developed	3	−3.28 (−6.03, −0.54)	0.019	0.0	0.602
Developing	3	−0.22 (−0.38, −0.07)	0.005	39.9	0.189
**Duration**					**0.854**
<12 weeks	2	−0.23 (−0.33, −0.13)	<0.001	0.0	0.336
12 weeks	3	−1.46 (−6.4, 3.48)	0.563	73.5	0.023
>12 weeks	1	−1.0 (−6.38, 4.38)	0.715	.	.
**Type of vitamin D**					**0.533**
Cholecalciferol	4	−1.31 (−4.95, 2.34)	0.482	60.2	0.056
Calcitriol	1	−0.18 (−0.32, −0.04)	0.014	.	.
22-oxa-calcitriol	1	−0.28 (−0.42, −0.14)	<0.001	.	.
**Dose of intervention**					**0.755**
≤50,000 IU/weekly	3	−0.25 (−0.47, −0.02)	0.031	65.6	0.055
>50,000 IU/weekly	3	0.30 (−3.12, 3.72)	0.864	29.6	0.242
**Type of control**					**0.626**
Placebo	3	−0.23 (−0.33, −0.13)	<0.001	0.0	0.606
Nothing	3	−1.46 (−6.4, 3.48)	0.563	73.5	0.023
Subgroup analysis for the effect of vitamin D supplementation on ESR.					
**Total**	**8**	**−4.08 (-4.67, -3.50)**	**<0.001**	**90.71**	**<0.001**
**Risk of bias**					**0.137**
Low	3	−4.15 (−4.74, −3.55)	<0.001	96.7	<0.001
Some concerns	1	−6.4 (−12.93, 0.13)	0.055	.	.
High	4	−0.51 (−4.28, 3.25)	0.789	71	0.016
**Country**					**<0.001**
Developed	4	3.28 (0.92, 5.63)	0.563	87.6	<0.001
Developing	4	−4.56 (−5.16, −3.96)	<0.001	74.2	0.009
**Duration**					**<0.001**
<12 weeks	2	−4.68 (−5.29, −4.07)	<0.001	0.0	0.700
12 weeks	4	4.91 (2.66, 1.17)	0.173	59.1	0.062
>12 weeks	2	−8.36 (−13.84, −2.88)	0.006	14.1	0.281
**Type of vitamin D**					**<0.001**
Ergocalciferol	1	−13 (−23.05, −2.95)	0.011	.	.
Cholecalciferol	5	3.71 (1.57, 5.84)	0.001	77.3	0.001
Calcitriol	1	−4.8 (−5.66, −3.94)	<0.001	.	.
22-oxa-calcitriol	1	−4.56 (−5.42, −3.70)	<0.001	.	.
**Dose of intervention**					**<0.001**
≤50,000 IU/weekly	4	−4.65 (−5.26, −4.04)	<0.001	0.0	0.767
>50,000 IU/weekly	4	3.97 (1.54, 6.03)	0.001	87.5	<0.001
**Type of control**					**<0.001**
Placebo	5	−4.71 (−5.32, −4.11)	<0.001	0.0	0.521
Nothing	3	5.82 (3.44, 8.21)	<0.001	0.0	0.401
Subgroup analysis for the effect of vitamin D supplementation on HAQ.					
**Total**	**5**	**−0.04 (-0.39, 0.31)**	**0.81**	**95.97**	**<0.001**
**Risk of bias**					**0.008**
low	3	−0.10 (−0.68, 0.48)	0.728	97.8	<0.001
Some concerns	1	0.24 (−0.14, 0.62)	0.216	.	.
high	1	−0.11 (−0.23, 0.01)	0.078	.	.
**Country**					**<0.001**
Developed	3	0.37 (0.28,0.47)	<0.001	0.0	0.485
Developing	2	−0.27 (−0.54, 0.01)	0.061	89.0	<0.001
**Duration**					**0.115**
<12 weeks	2	−0.35 (−0.78, 0.08)	0.112	91.3	0.001
12 weeks	2	0.14 (−0.34, 0.62)	0.578	97.3	<0.001
>12 weeks	1	0.24 (−0.14, 0.62)	0.216	.	.
**Type of vitamin D**					**<0.001**
Ergocalciferol	1	0.24 (−0.14, 0.62)	0.216	.	.
Cholecalciferol	2	0.14 (−0.34, 0.62)	0.578	97.3	<0.001
Calcitriol	1	−0.13 (−0.31, 0.05)	0.151	.	.
22-oxa-calcitriol	1	−0.57 (−0.75, −0.39)	<0.001	.	.
**Dose of intervention**					**<0.001**
≤50,000 IU/weekly	4	−0.17 (−0.44, 0.09)	0.206	87.3	<0.001
>50,000 IU/weekly	1	0.38 (0.28, 0.48)	<0.001	.	.
**Type of control**					
Placebo	4	−0.17 (−0.44, 0.09)	0.206	87.3	<0.001
Nothing	1	0.38 (0.28, 0.48)	<0.001	.	.
Subgroup analysis for the effect of vitamin D supplementation on VAS.					
**Total**	**6**	**0.17 (-0.77, 1.1)**	**0.73**	**98.65**	**<0.001**
**Risk of bias**					**0.323**
low	3	0.14 (−1.15, 1.42)	0.835	99.4	<0.001
Some concerns	1	1.45 (−0.26, 3.16)	0.097	.	.
high	2	−0.23 (−1.73, 1.27)	0.764	87.8	0.004
**Country**					**<0.001**
Developed	2	2.13 (1.83, 2.43)	<0.001	0.0	0.430
Developing	4	−0.73 (−1.02, −0.43)	<0.001	81.2	<0.001
**Duration**					**0.013**
<12 weeks	2	−0.86 (−1.04, −0.67)	<0.001	74.4	0.048
12 weeks	3	0.59 (−1.44, 2.62)	0.569	97.3	<0.001
>12 weeks	1	1.45 (−0.77, 1.1)	0.97	.	.
**Type of vitamin D**					**0.005**
Ergocalciferol	1	1.45 (−0.26, 3.16)	0.097	.	.
Cholecalciferol	3	0.59 (−1.44, 2.62)	0.569	97.3	<0.001
Calcitriol	1	−0.76 (−0.90, −0.62)	<0.001	.	.
22-oxa-calcitriol	1	−0.95 (−1.08, −0.82)	<0.001	.	.
**Dose of intervention**					**0.466**
≤50,000 IU/weekly	4	−0.55 (−0.93, −0.17)	0.004	86.7	<0.001
>50,000 IU/weekly	2	0.60 (−2.47, 3.67)	0.701	98.5	<0.001
**Type of control**					**0.466**
Placebo	4	−0.55 (−0.93, −0.17)	0.004	86.7	<0.001
Nothing	2	0.60 (−2.47, 3.67)	0.701	98.5	<0.001
Subgroup analysis for the effect of vitamin D supplementation on serum vitamin D.					
**Total**	**7**	**12.69 (1.80, 23.59)**	**0.02**	**99.72**	**<0.001**
**Risk of bias**					**0.291**
low	3	4.27 (1.15, 7.4)	0.007	94.6	<0.001
Some concerns	1	3.00 (−10.67, 16.67)	0.677	.	.
high	3	23.46 (−0.51, 47.43)	0.055	99.6	<0.001
**Country**					**0.884**
Developed	4	12.6 (5.84, 19.35)	<0.001	91.6	<0.001
Developing	3	13.83 (−1.36, 29.02)	0.074	99.8	<0.001
**Duration**					0.161
<12 weeks	2	1.55 (1.00, 2.09)	<0.001	0.0	0.678
12 weeks	4	20.27 (0.98, 39.56)	0.039	99.6	<0.001
>12 weeks	1	3.00 (−10.67, 16.67)	0.677	.	.
**Type of vitamin D**					**0.280**
Ergocalciferol	1	3.00 (−10.67, 16.67)	0.677	.	.
Cholecalciferol	4	20.27 (0.98, 39.56)	0.039	99.6	<0.001
Calcitriol	1	1.43 (0.66, 2.20)	<0.001	.	.
22-oxa-calcitriol	1	1.66 (0.89, 2.43)	<0.001	.	.
**Dose of intervention**					**0.217**
≤50,000 IU/weekly	5	6.19 (1.09, 11.28)	0.017	98.3	<0.001
>50,000 IU/weekly	2	28.94 (−6.79, 64.67)	0.112	99.8	<0.001
**Type of control**					**0.037**
Placebo	4	1.6 (1.06, 2.13)	<0.001	0.0	0.499
Nothing	3	25.30 (3.00, 47.61)	0.026	99.7	<0.001

Abbreviations: WMD, weighted mean difference; CI, confidence interval; DAS-28, disease activity score-28; CRP, C-reactive protein; ESR, erythrocyte sedimentation rate; HAQ, the health assessment questionnaire; VAS, visual analog scale.

### 3.13 Grading of evidence

Table S4 presents the comprehensive GRADE evidence for each outcome. The findings indicate moderate certainty of the evidence for all outcomes, except for serum vitamin D, which has high certainty of the evidence.

## 4 Discussion

The current study's findings demonstrate that vitamin D supplementation significantly reduced serum levels of CRP and ESR concentration. We also discovered a remarkable improvement in DAS-28 scores and serum vitamin D levels. However, any significant changes in HAQ and VAS scores were not found. A moderate certainty of evidence for DAS28, ESR, CRP, HAQ, and VAS and a high certainty of evidence for serum vitamin D were obtained.

Vitamin D, which is in the fat-soluble vitamins group, is missing in terms of nutritional value in many people worldwide and has been evaluated for its importance during the last few decades in preventing and treating several diseases [43]. In patients with RA, several epidemiological studies have demonstrated lower vitamin D levels in RA patients compared to the control group. Gopal et al., in a cohort study, found that more than 63 % of patients with RA had vitamin D deficiency [44]. Also, Nakayama et al. showed that more than 76 % of patients suffering from RA had low serum vitamin D levels [45]. Therefore, several interventional studies have been designed and implemented in recent years to examine the effect of vitamin D supplementation on clinical symptoms and biochemical biomarkers in these patients.

Due to the significant reduction in the concentration of CRP and ESR, a significant anti-inflammatory impact of vitamin D was shown in the current study. In RA pathogenesis, inflammation is a significant component [46]. Exacerbation of inflammation through several mechanisms causes further progression of the disease, more pain, and lower quality of life in these patients [47]. Vitamin D has beneficial effects in preventing inflammation, as shown in several studies. Moslemi et al., in an umbrella meta-analysis of 32 meta-analysis studies, identified an important decrease in serum level of CRP was achieved by the supplementation of vitamin D [48]. In the primary studies included in the current study, researchers used different doses of vitamin D. The results of the subgroup analysis demonstrate that in all of our outcomes, the intervention dose lower than 50,000 IU weekly had more significant or better impacts. Regarding these results, in a study by Moslemi et al., the subgroup analysis showed that the most anti-inflammatory implications of vitamin D were in the dose below 3500 IU a day, and with increasing the dose above 3500 IU, the power of vitamin D in exerting anti-inflammatory effects decreased [48]. Vitamin D and its active form have demonstrated powerful anti-inflammatory effects by inhibiting the pro-inflammatory transcription factor Nuclear Factor Kappa B (NF-κB) [49]. This is achieved through the activation of NF-κB inhibitors, specifically IκB-α, which helps to reduce inflammation [50,51]. The presence of NF-κB can enhance the effects of the Signal transducer and activator of transcription-3 (STAT3) and the production of CRP [52]. Research has shown that vitamin D supplementation can lower CRP levels by inhibiting both the NF-κB and STAT3 signaling pathways [52,53].

In the present study, vitamin D supplementation caused a significant improvement in the DAS-28 scores. The DAS-28 is known as a measurement tool for disease activity in RA [54]. DAS-28 helps clinicians evaluate the severity of the disease and monitor treatment effectiveness [55]. The DAS-28 score is calculated using a specific formula incorporating the swollen and tender joints counts, the patient's global assessment score, and the acute phase reactant level [56]. It has been stated that DAS-28 is a valid tool for evaluating the activity of RA [27]. Previous research showed an opposite relationship between serum levels of 25(OH)D and DAS-28 scores, which agrees with our findings [57,58].

Various indices are used to evaluate the pain level as one of the main symptoms of RA, which are mainly based on scores, and which VAS pain score is one of the most reliable indicators [59]. Literature shows that vitamin D supplementation may help manage and relieve musculoskeletal pain and neuromuscular coordination, especially in people with insufficient vitamin D levels [60,61]. Nevertheless, in line with our results, Schreuder et al. in a study that evaluates the effects of vitamin D supplementation on musculoskeletal pain showed insignificant changes in VAS score [62].

One special result in subgroup analysis revealed that both developed and developing countries have significant changes separately, improvement in developing countries and deterioration in developed countries (p-value <0.001 for both). Assessing other outcomes showed except for HAQ, all measurements improved significantly in the developing subgroups. It seems developing countries benefit more from vitamin D supplementation, which could help them improve symptoms and inflammatory markers. A hypothesis for this relies on the more significant inverse relationship between serum 25-hydroxyvitamin D level and disease activity index in RA patients of developing countries than in developed countries [63]. In fact, RA patients with low serum vitamin D levels experience worse disease activity in developing countries, which may explain the better results in this subgroup of patients.

Vitamin D metabolites interact with the vitamin D receptor (VDR) in human chondrocyte cultures, thereby regulating the transcription of various genes related to chondrocyte metabolism [64]. This protective action also modifies the expression of particular matrix metalloproteinases and induces the formation of proteoglycan and collagen. Moreover, some evidence showed the expression of VDR human rheumatoid synovium and from macrophages, chondrocytes, and synoviocytes in a junction between the cartilage and the pannus, whereas VDR expression is absent in the control group [[65], [66], [67]]. Experts have proposed that vitamin D may have a notable impact in reducing musculoskeletal pain due to VDR in the skeletal muscle cells [68]. The evaluations on VDR knock-out mice revealed that the muscle fibers were of a smaller and more varied size, yet myocyte differentiation still occurred as expected [61,69]. According to several recent studies, inflammation may not be the only cause of the chronic pain that RA patients continue to suffer even after getting the anti-inflammatory agents, and the occurrence of that may also be influenced by chronic hypersensitivity [70,71]. Studies have revealed that both at joint and nonjoint sites, RA patients’ pressure pain thresholds are lower (they are more sensitive to pain) than healthy volunteers [72,73]. The lack of vitamin D, a neuroactive nutrient, may be an unrecognized cause of nociception and reduced neuromuscular performance in people with chronic pain [74,75].

Another point worth mentioning is subgroup analysis by the type of placebo. For CRP, ESR, and VAS, there were improvements in studies that used a placebo for the control group subgroup, and serum vitamin D was increased more in the placebo subgroup. Since studies using a placebo show more reliable results, owing to a lower risk of bias, the results of this study can strengthen significantly in the CRP, ESR, and VAS. In the end, we should mention that due to the minimal clinically important differences (MCID) cut point, which is the smallest change in a treatment outcome that an individual patient would identify as important and would indicate a change in the patient's management, is mentioned in Table S5 for each outcome. From all outcomes, only serum vitamin D showed a clinically significant improvement compared to others. It makes it clear that vitamin D may not be a good choice in order to control the symptoms and inflammation in RA and could not be a therapeutic agent for their disease. Supplementation of vitamin D can be considered to increase the serum level of this vital vitamin in patients with RA and also assist in managing the symptoms besides other therapeutic agents.

The current study had several strengths, among which we can point out the detailed systematic search, the search of various databases, and the lack of language restrictions in the search process. We also run a detailed subgroup analysis to address the source of heterogeneity. However, the results of this study can be influenced by some limitations. A small sample size often limits the studies in this area, and the results are usually highly varied, with a lack of examination of the effect of sun exposure and the RA stage (early or late stage) in the included primary studies. Moreover, some studies did not report the crude baseline and endpoint values and just reported the mean change. Thus, we were not able to assess these points, and that may result in a limitation in the absolute effects of vitamin D effects on our outcomes. Another noteworthy point is that significant heterogeneity was encountered, perhaps due to various regimens, doses, duration, center settings, and populations enrolled. A high risk of bias is another point that should be considered since it may affect the results. Furthermore, the effect on many occasions was assessed by very few studies; thus, the evidence to support it is low.

## 5 Conclusion

Significant effects on the levels of ESR, CRP, and DAS-28 score of patients with RA have been demonstrated by the use of vitamin D supplements in the intervention groups compared to the control groups. Since the optimal dosage and length of treatment are still unknown in these patients, further research is needed to ascertain the optimal dosage of this supplementation.

## CRediT authorship contribution statement

**Mahsa Ranjbar:** Writing – original draft, Software, Methodology, Investigation, Formal analysis, Data curation, Conceptualization. **Mehran Rahimlou:** Writing – original draft. **Maryam Fallah:** Data curation. **Kurosh Djafarian:** Methodology, Investigation. **Hamed Mohammadi:** Validation, Supervision, Methodology, Investigation, Conceptualization.

## Availability of data and materials

The datasets used during the current study are available from the corresponding author upon reasonable request.

## Ethics approval and consent to participate

Not applicable.

## Consent for publication

Not applicable.

## Support

This study was supported by the Students’ Scientific Research Center of 10.13039/501100004484Tehran University of Medical Sciences, Iran (code: 1402-2-125-66889), and they have no role in the design and preparation of the study.

## Declaration of competing interest

The authors declare that they have no known competing financial interests or personal relationships that could have appeared to influence the work reported in this paper.
