# Supplementary material for: Effects of vitamin D supplementation in patients with rheumatoid arthritis: A systematic review and meta-analysis
Source: Heliyon. 2025 Feb 4;11(3):e42463. doi: 10.1016/j.heliyon.2025.e42463 (PMC11849653; doi:10.1016/j.heliyon.2025.e42463)
Supplement: Multimedia component 1 [file mmc1.docx]

**Effects of Vitamin D Supplementation in Patients with Rheumatoid Arthritis: A Systematic Review and Meta-Analysis**

Mahsa Ranjbar^1,2^, Mehran Rahimlou^3^, Maryam Fallah^1^, Kurosh Djafarian^1,4^, Hamed Mohammadi^1^

^1^*Department of Clinical Nutrition, School of Nutritional Sciences and Dietetics, Tehran University of Medical Science, Tehran, Iran*

*^2^Students’ Scientific Research Center, Tehran University of Medical Sciences, Tehran, Iran*

*^3^Department of Nutrition, Faculty of Medicine, Zanjan University of Medical Sciences, Zanjan, Iran*

*^4^Neuroscience Institute, Sports Medicine Research Center, Tehran University of Medical Sciences, Tehran, Iran*

Supplementary data including Supplementary Tables 1-5, and Figures 1-3

| **Table S1.** Search strategies including the key terms and the queries for each database. | |
| --- | --- |
| **Database**  **5/12/2024** | **key terms and the queries** |
| PubMed  (n=1,340) | ("arthritis, rheumatoid"[MeSH Terms] OR "arthritis rheumatoid"[Title/Abstract] OR "rheumatoid arthritis"[Title/Abstract] OR "RA"[Title/Abstract]) AND ("vitamin d"[MeSH Terms] OR "vitamin d"[Title/Abstract] OR "calciferol"[Title/Abstract] OR "cholecalciferol"[Title/Abstract] OR "cholecalciferol"[MeSH Terms] OR "25 hydroxyvitamin d"[Title/Abstract] OR "25 hydroxycholecalciferol"[Title/Abstract] OR "calcidiol"[Title/Abstract] OR "calcitriol"[Title/Abstract] OR "calcitriol"[MeSH Terms] OR "1 25 dihydroxycholecalciferol"[Title/Abstract]) |
| Web of Science (ISI)  (n=2,056) | "#1 "arthritis, rheumatoid" (Topic) or "arthritis rheumatoid" (Topic) or "rheumatoid arthritis" (Topic) or "RA" (Topic)  #2 "vitamin d" (Topic) or "calciferol" (Topic) or "cholecalciferol" (Topic) or "25 hydroxyvitamin d" (Topic) or "25 hydroxycholecalciferol" (Topic) or "calcidiol" (Topic) or "calcitriol" (Topic) or "1 25 dihydroxycholecalciferol" (Topic)  #1 AND #2 |
| Scopus  (n=3,502) | ( ( TITLE-ABS-KEY ( "vitamin d" ) OR TITLE-ABS-KEY ( "calciferol" ) OR TITLE-ABS-KEY ( "cholecalciferol" ) OR TITLE-ABS-KEY ( "25 hydroxyvitamin d" ) OR TITLE-ABS-KEY ( "25 hydroxycholecalciferol" ) OR TITLE-ABS-KEY ( "calcidiol" ) OR TITLE-ABS-KEY ( "calcitriol" ) OR TITLE-ABS-KEY ( "1 25 dihydroxycholecalciferol" ) ) ) AND ( ( ( TITLE-ABS-KEY ( "arthritis, rheumatoid" ) OR TITLE-ABS-KEY ( "arthritis rheumatoid" ) OR TITLE-ABS-KEY ( "rheumatoid arthritis" ) OR TITLE-ABS-KEY ( "RA" ) ) ) ) |

| **Table S2**: Reason for exclusion of retrieved articles | |
| --- | --- |
| References | Reason for exclusion |
| 1. Alizadeh M, Astill J, Alqazlan N, Shojadoost B, Taha-Abdelaziz K, Bavananthasivam J, et al. In ovo co-administration of vitamins (A and D) and probiotic lactobacilli modulates immune responses in broiler chickens. Poult Sci. 2022;101(4):101717.  2. Gajra A, Akbar SA, Din NU. Management of Lung Cancer in the Elderly. Clinics in Geriatric Medicine. 2016;32(1):81-95.  3. da Silveira KL, da Silveira LL, Thorstenberg ML, Cabral FL, Castilhos LG, Rezer JF, et al. Free and nanoencapsulated vitamin D3 : effects on E-NTPDase and E-ADA activities in an animal model with induced arthritis. Cell Biochem Funct. 2016;34(4):262-73.  4. Dillitzer N, Becker N, Kienzle E. Intake of minerals, trace elements and vitamins in bone and raw food rations in adult dogs. Br J Nutr. 2011;106 Suppl 1:S53-6.  5. An J, Yuan Q, Wang C, Liu L, Tang K, Tian HY, et al. Differential display of proteins involved in the neural differentiation of mouse embryonic carcinoma P19 cells by comparative proteomic analysis. Proteomics. 2005;5(6):1656-68.  6. Baas D, Prüfer K, Ittel ME, Kuchler-Bopp S, Labourdette G, Sarliève LL, et al. Rat oligodendrocytes express the vitamin D(3) receptor and respond to 1,25-dihydroxyvitamin D(3). Glia. 2000;31(1):59-68.  7. Ametaj BN, Nonnecke BJ, Horst RL, Beitz DC. Effects of retinoic acid and 1,25-dihydroxyvitamin D3 on IFN-gamma secretion by mononuclear leukocytes from nulliparous and postparturient dairy cattle. Int J Vitam Nutr Res. 2000;70(3):92-101.  8. Alvarez-Dolado M, González-Sancho JM, Navarro-Yubero C, García-Fernández LF, Muñoz A. Retinoic acid and 1,25-dihydroxyvitamin D3 inhibit tenascin-C expression in rat glioma C6 cells. J Neurosci Res. 1999;58(2):293-300.  9. Cantorna MT, Hayes CE, DeLuca HF. 1,25-dihydroxycholecalciferol inhibits the progression of arthritis in murine models of human arthritis. Journal of Nutrition. 1998;128(1):68-72.  10. Chentoufi J, Marie PJ. Interactions between retinoic acid and 1,25(OH)2D in mouse immortalized osteoblastic C1 cells. Am J Physiol. 1994;266(5 Pt 1):C1247-56. | Studies on animals |
| 11. Suzuki T, Nakamura Y, Kato H. Calcium and vitamin D supplementation with 3-year denosumab treatment is beneficial to enhance bone mineral density in postmenopausal patients with osteoporosis and rheumatoid arthritis. Ther Clin Risk Manag. 2019;15:15-22.  12. Racovan M, Walitt B, Collins CE, Pettinger M, Parks CG, Shikany JM, et al. Calcium and vitamin D supplementation and incident rheumatoid arthritis: the Women's Health Initiative Calcium plus Vitamin D trial. Rheumatol Int. 2012;32(12):3823-30.  13. Buckley LM, Leib ES, Cartularo KS, Vacek PM, Cooper SM. Calcium and vitamin D-3 supplementation prevents bone loss in the spine secondary to low-dose corticosteroids in patients with rheumatoid arthritis - A randomized, double-blind, placebo-controlled trial. Annals of Internal Medicine. 1996;125(12):961-+. | Combination of vitamin D and calcium |
| 4. Talbi S, Aradoini N, Salia K, Abourazzak FE, Harzy T. VITAMIN D AND ITS RELATIONSHIP WITH CLINICAL AND LABORATORY PARAMETERS IN PATIENTS WITH RHEUMATOID ARTHRITIS. Annals of the Rheumatic Diseases. 2015;74:699-.  15. Varenna M, Manara M, Cantatore FP, Del Puente A, Di Munno O, Malavolta N, et al. Determinants and effects of vitamin D supplementation on serum 25-hydroxy-vitamin D levels in patients with rheumatoid arthritis. Clin Exp Rheumatol. 2012;30(5):714-9. | Observational studies |
| 16. Aung K. VITAMIN D SUPPLEMENTATION ON DISEASE ACTIVITY IN PATIENTS WITH RHEUMATOID ARTHRITIS. Annals of the Rheumatic Diseases. 2020;79:1386-.  17. Adami G, Rossini M, Bogliolo L, Cantatore FP, Varenna M, Malavolta N, et al. An exploratory study on the role of vitamin D supplementation in improving pain and disease activity in rheumatoid arthritis. Mod Rheumatol. 2019;29(6):1059-62.  18. Gopal K, Thevarajah M, Ng CM, Raja J. EFFECTS OF VITAMIN D ON DISEASE ACTIVITY, FUNCTIONAL DISABILITY AND SERUM INTERLEUKIN-6 IN RHEUMATOID ARTHRITIS. Annals of the Rheumatic Diseases. 2018;77:1747-8.  19. Chandrashekara S, Patted A. Role of vitamin D supplementation in improving disease activity in rheumatoid arthritis: An exploratory study. Int J Rheum Dis. 2017;20(7):825-31.  20. Yang J, Liu L, Zhang Q, Li M, Wang J. Effect of vitamin D on the recurrence rate of rheumatoid arthritis. Exp Ther Med. 2015;10(5):1812-6.  21. von Restorff C, Bischoff-Ferrari HA, Theiler R. High-dose oral vitamin D3 supplementation in rheumatology patients with severe vitamin D3 deficiency. Bone. 2009;45(4):747-9.  22. Andjelkovic Z, Vojinovic J, Pejnovic N, Popovic M, Dujic A, Mitrovic D, et al. Disease modifying and immunomodulatory effects of high dose 1α(OH) D3 in rheumatoid arthritis patients. Clinical and Experimental Rheumatology. 1999;17(4):453-6. | Do not have a control group |
| 23. Kwon OC, Oh JS, Park MC, Kim YG. Effect of Vitamin D Supplementation on Bone Mineral Density in Rheumatoid Arthritis Patients With Osteoporosis. Front Med (Lausanne). 2020;7:443.  24. Anno S, Inui K, Okano T, Mamoto K, Sugioka Y, Tada M, et al. THE EFFECT OF CONCOMITANT TYPE OF VITAMIN D, BIOLOGICAL DMARDS AND DISEASE ACTIVITY FOR THERAPEUTIC EFFECT OF DENOSUMAB IN OSTEOPOROSIS PATIENTS WITH RHEUMATOID ARTHRITIS. Annals of the Rheumatic Diseases. 2018;77:456-.  25. Dehghan A, Rahimpour S, Soleymani-Salehabadi H, Owlia MB. Role of vitamin D in flare ups of rheumatoid arthritis. Z Rheumatol. 2014;73(5):461-4.  26. Gopinath K, Danda D. Supplementation of 1,25 dihydroxy vitamin D3 in patients with treatment naive early rheumatoid arthritis: a randomised controlled trial. Int J Rheum Dis. 2011;14(4):332-9.  27. Den Uyl D, Geusens PPMM, Van Berkum FNR, Houben HHML, Jebbink MC, Lems WF. Patient preference and acceptability of calcium plus vitamin D3 supplementation: A randomised, open, cross-over trial. Clinical Rheumatology. 2010;29(5):465-72. | Do not report related outcomes |
| 28. Manson JE, Bassuk SS, Lee IM, Cook NR, Albert MA, Gordon D, et al. The VITamin D and OmegA-3 TriaL (VITAL): rationale and design of a large randomized controlled trial of vitamin D and marine omega-3 fatty acid supplements for the primary prevention of cancer and cardiovascular disease. Contemp Clin Trials. 2012;33(1):159-71. | Protocol study |

**Table S3.** Cochrane Risk of Bias Assessment.

| Study | Random Sequence Generation | Allocation concealment | Blinding of participants and personnel | Blinding of outcome assessment | Incomplete outcome data | Selective outcome reporting | Other sources of bias | Overall quality |
| --- | --- | --- | --- | --- | --- | --- | --- | --- |
| Brohult et al.  (Sweden; 1973) | L | U | L | U | L | L | U | H |
| Buondonno et al.  (Italy; 2017) | L | L | L | L | L | L | L | L |
| El-Banna et al.  (Egypt; 2020) | L | U | H | H | U | L | U | H |
| Hansen et al.  (USA; 2014) | L | U | L | U | L | L | L | Some concerns |
| Li et al.  (China; 2018) | L | L | L | U | L | L | L | L |
| Salesi et al.  (Iran; 2012) | L | U | L | U | L | L | U | H |
| Soubrier et al.  (France; 2018) | L | U | L | U | L | L | L | Some concerns |
| Alotalibi et al, (Egypt; 2021) | L | H | H | H | L | L | L | H |
| Chandrashekara et al. (India; 2015) | L | H | H | H | L | L | L | H |
| Mukherjee et al. (India; 2019) | L | H | H | H | L | L | L | H |
| Elfituri  (The UK; 2024) | L | U | U | U | L | L | U | H |

**Abbreviations:** L, low risk of bias; H, high risk of bias; U, unclear risk of bias.

**Table S4.** Certainty of evidence using the GRADE approach.

**Question:** [intervention] compared to [comparison] for [health problem and/or population]

| **Certainty assessment** | | | | | | | **№ of patients** | | **Effect** | | **Certainty** | **Importance** |
| --- | --- | --- | --- | --- | --- | --- | --- | --- | --- | --- | --- | --- |
| **№ of studies** | **Study design** | **Risk of bias** | **Inconsistency** | **Indirectness** | **Imprecision** | **Other considerations** | **[intervention]** | **[comparison]** | **Relative (95% CI)** | **Absolute (95% CI)** |  |  |
| **DAS-28 (assessed with: Questionare)** | | | | | | | | | | | | |
| 9 | randomised trials | not serious^a^ | not serious | not serious | serious^b^ | none | 364 | 327 | - | MD **0.83 lower** (1.38 lower to 0.28 lower) | ⨁⨁⨁◯ Moderate |  |
| **CRP (assessed with: Blood test)** | | | | | | | | | | | | |
| 6 | randomised trials | not serious^c^ | not serious | not serious | serious^d^ | none | 414 | 379 | - | MD **0.24 lower** (0.45 lower to 0.03 lower) | ⨁⨁⨁◯ Moderate |  |
| **ESR (assessed with: Blood test)** | | | | | | | | | | | | |
| 8 | randomised trials | not serious^e^ | not serious | not serious | serious^f^ | none | 488 | 452 | - | MD **4.08 lower** (4.67 lower to 3.5 lower) | ⨁⨁⨁◯ Moderate |  |
| **HAQ (assessed with: Questionare)** | | | | | | | | | | | | |
| 5 | randomised trials | not serious^g^ | not serious | not serious | serious^h^ | none | 304 | 305 | - | MD **0.04 lower** (0.39 lower to 0.31 higher) | ⨁⨁⨁◯ Moderate |  |
| **VAS (assessed with: questionare)** | | | | | | | | | | | | |
| 6 | randomised trials | not serious^i^ | not serious | not serious | serious^j^ | none | 398 | 388 | - | MD **0.56 lower** (0.92 lower to 0.19 lower) | ⨁⨁⨁◯ Moderate |  |
| **serum vitamin d (assessed with: blood test)** | | | | | | | | | | | | |
| 7 | randomised trials | not serious^k^ | not serious | not serious | not serious | none | 446 | 408 | - | MD **12.69 higher** (1.8 higher to 23.59 higher) | ⨁⨁⨁⨁ High |  |

**CI,** confidence interval; **MD,** mean difference; **CRP**, C-reactive protein; **ESR**, erythrocyte sedimentation rate; **HAQ**, The Health Assessment Questionnaire; **VAS**, visual analog scale.

**Explanations**

a. because 2 studies have some concerns and 6 studies have a high risk of bias, downgraded. However, the result in the low risk of bias subgroup was like the total results. Upgraded.

b. serious inconsistency because the point estimate was lower than MCID (WMD: -0.83, MCID: 1.2). downgraded.

c. because 2 studies have some concerns and 2 studies have a high risk of bias, downgraded. However, the result in the low risk of bias subgroup was like the total results. Upgraded.

d. serious inconsistency because the point estimate was lower than MCID (WMD: -0.24, MCID: 0.5). downgraded.

e. because 1 study has some concerns and 4 studies have a high risk of bias, downgraded. However, the result in the low risk of bias subgroup was like the total results. Upgraded.

f. serious inconsistency because the point estimate was lower than MCID (WMD: -4.67, MCID: 13.23). downgraded.

g. because 1 study has some concerns and 1 study has a high risk of bias, downgraded. However, the result in the low-risk of-bias subgroup was like the total results. Upgraded.

h. serious inconsistency because the point estimate was lower than MCID (WMD: -0.04, MCID: 0.68). downgraded.

i. because 1 study has some concerns and 2 studies have a high risk of bias, downgraded. However, the result in the low-risk of-bias subgroup was like the total results. Upgraded

.j. serious inconsistency because the point estimate was lower than MCID (WMD: -0.77, MCID: 2.4). downgraded.

k. because 1 study has some concerns and 3 studies have a high risk of bias, downgraded. However, the result in the low-risk of-bias subgroup was like the total results. Upgraded

| Table S5. Minimal clinically important differences (MCID) for outcomes^1^. | |
| --- | --- |
| Variable (ref.) | MCID (unit) |
| Disease activity score-28 (DAS-28) (1) | 1.2 |
| Erythrocyte sedimentation rate (ESR) (2) | 13.23 mm/h |
| C-reactive protein (CRP) (3) | 0.5 mg/l |
| Visual analog scale (VAS) (2) | 2.4 |
| Health Assessment Questionnaire (HAQ) (4) | 0.68 |
| Serum vitamin D (2) | 9.25 ng/ml |
| ^1^ Numbers in parenthesis refer to supplemental references. | |

**Supplemental Figures**

Figure S1. **Risk of bias across included studies. Studies were assessed as “Low risk of bias” if the overall study design and conduct had no substantial deviations that were likely to bias true effect estimate, “Unclear risk of bias” if sufficient information was not provided to assess the risk of bias, and “High risk of bias” if the design and conduct of the study was likely to have substantial influence on true effect estimate.**

**Figure 2-A Figure 2-B**

**Figure 2-C Figure 2-D**

**Figure 2-E Figure 2-F**

Figure S2 **Funnel plots assessing publication bias and effect of small studies for (A) disease activity score-28 (DAS-28), (B) C-reactive protein (CRP) (C) erythrocyte sedimentation rate (ESR), (D) health assessment questionnaire (HAQ), (E) visual analog scale, and (F) serum vitamin D.**

**Figure 3-A Figure 3-B**

**Figure 3-C Figure 3-D**

**Figure 3-E Figure 3-F**

Figure S3 **Forest plots of sensitivity analysis with leave-one-out meta-analysis (A) disease activity score-28 (DAS-28), (B) C-reactive protein (CRP) (C) erythrocyte sedimentation rate (ESR), (D) health assessment questionnaire (HAQ), (E) visual analog scale, and (F) serum vitamin D. Results are expressed as mean difference (MD) with 95%CI for the remaining studies after excluding one study.**

1. Fransen J, Van Riel P. The Disease Activity Score and the EULAR response criteria. Clinical and experimental rheumatology. 2005;23(5):S93.

2. Norman GR, Sloan JA, Wyrwich KW. Interpretation of changes in health-related quality of life: the remarkable universality of half a standard deviation. Medical care. 2003:582-92.

3. Goldenberg JZ, Day A, Brinkworth GD, Sato J, Yamada S, Jönsson T, et al. Efficacy and safety of low and very low carbohydrate diets for type 2 diabetes remission: systematic review and meta-analysis of published and unpublished randomized trial data. bmj. 2021;372.

4. Behrens F, Koehm M, Schwaneck EC, Schmalzing M, Gnann H, Greger G, et al. Use of a “critical difference” statistical criterion improves the predictive utility of the Health Assessment Questionnaire-Disability Index score in patients with rheumatoid arthritis. BMC Rheumatology. 2019;3(1):51.

**Supplementary references:**
